# COVID-19 pandemic: Challenges and advances in the Physical Therapy, Speech-Language-Hearing Science, and Occupational Therapy undergraduate programs in Brazil

**DOI:** 10.6061/clinics/2020/e2490

**Published:** 2020-11-25

**Authors:** Alessandra G. Samelli, Carla G. Matas, Naomi K. Nakagawa, Talita N Rossi da Silva, Milton A. Martins, Sílvia Maria Amado João

**Affiliations:** I Departamento de Fisioterapia, Fonoaudiologia e Terapia Ocupacional, Faculdade de Medicina (FMUSP), Universidade de Sao Paulo, Sao Paulo, SP, BR; II Departamento de Clinica Medica, Faculdade de Medicina (FMUSP), Universidade de Sao Paulo, Sao Paulo, SP, BR

Due to the COVID-19 pandemic, educational systems all over the world have been facing unprecedented challenges. The World Bank reported that an estimated 1.4 billion students were away from school in more than 156 countries (1). Schools and universities implemented, with varying degrees of success, various forms of distance learning (DL) and remote learning (RL), in an attempt to diminish the educational impacts of the pandemic.

The guiding principles of RL are the same as those of the teaching and learning process in-person. This strategy is similar to DL in that the teacher and the students are not present in the same room, and that it is developed through technology, which is not the only option in DL. Moreover, DL results from planning, choosing, and developing careful and systematic instructional designs, presupposing the atemporal support of tutors, greater flexibility for students to organize their study hours, and the possibility of carrying out synchronous, asynchronous, and mixed activities—especially asynchronous ones (2).

In the context of the pandemic, RL was adopted as a means of continuing undergraduate courses. This strategy is referred to as emergency RL. Since there was insufficient time for careful planning, a temporary and fast change in the teaching model was adopted to adjust to the crisis. Hence, emergency RL involves fully remote teaching solutions that provide instruction, which would otherwise have taken place in-person—a format that will recommence as soon as the crisis or emergency has subsided. Thus, the effort is to carry on the courses following their usual timetable, with asynchronous and synchronous activities. The latter maintains a format closer to the lectures that would normally have taken place in traditional in-person learning (2). 

In March 2020, the Ministry of Education (MEC) authorized in-person classes throughout the Brazilian education system to be replaced with online classes for the duration of the COVID-19 pandemic (3). The sudden suspension of in-person classes was a preventive measure to avoid the dissemination of COVID-19. Therefore, academic learning activities had to migrate immediately to the virtual environment. Even though online teaching was not a new concept altogether, it was a change from traditional in-person teaching. The shift brought about significant challenges both to educators and students (4-8). This situation was similar in the case of the Physical Therapy, Speech-Language-Hearing Science, and Occupational Therapy undergraduate programs at the Medical School of the University of São Paulo (FMUSP).

Throughout this time, the students in the three programs were consulted regarding their needs that arose as a result of the pandemic and online emergency RL. Besides the new educational demands imposed by the pandemic, other factors (such as social isolation, change in routine, increase in domestic demands, and the concern with viral contamination) also interfered with the teaching and learning process. Other pandemic-related difficulties faced by the students included, but were not limited to, their family’s tighter financial and socio-economic situation, sick and dying friends, neighbors, and relatives, lack of an appropriate home environment to study, and lack of or difficulty with technological resources to follow the online RL (internet connection and personal computer).

Similar studies based in other countries highlight that the transition from in-person to RL requires a gradual and adaptive process for both educators and students. An unexpected fully online learning method, such as that implemented due to the pandemic, inevitably has weaknesses and risks. Students feel more distant from educators and learners are less focused while in the virtual environment (5,9).

Another study investigated the perception of medical students at an American university and verified that, from the students’ standpoint, substituting in-person learning with RL had negative and positive effects on their learning. The negative effects were mainly related to the loss of practical experience and strong feelings of anxiety and isolation. On the other hand, the positive aspects of RL included greater flexibility, opportunities to explore different learning resources, and time to focus on well-being. The authors concluded the study emphasizing the need to develop both sustainable remote academic curricula and professors’ fluency in DL formats and technologies (6).

Countries with advanced technology have efficient e-learning systems and online medical education. However, this is not the case in many low- and middle-income countries. For instance, a study conducted in Pakistan during the pandemic, when in-person classes had been suspended, evidenced various challenges faced by professors and students in the Faculty of Medicine, including untrained faculty, lack of institutional technical and technological support, internet connectivity issues, difficulties maintaining students’ involvement, difficulties with online assessments, and problems understanding the dynamics of online RL. All parties must cooperate and take an active role in overcoming these challenges (10).

In this sense, significant effort was made to resolve a similar situation at FMUSP and to minimize barriers to learning brought about by the pandemic. For example, the FMUSP provided internet SIM cards and Chromebooks to students who had trouble accessing the internet (11). Moreover, the Student Support Center, the Student Psychological Assistance Group at FMUSP, the Medical Education Development Center, and the Coordinators of the three undergraduate programs (Speech-Language-Hearing Science, Physical Therapy, and Occupational Therapy), in partnership with the FMUSP Graduation Committee, developed a continuous work plan to consult with and monitor students and the difficulties they experienced. These difficulties were resolved quickly in various instances.

The Covid-19 pandemic posed countless difficulties to educators as well. Like the students, they experienced restrictions and sanitary, social, economic, and financial concerns that emerged or worsened with the pandemic. From this perspective, educators took on other challenges with emergency RL, such as adapting learning content and changing classroom dynamics and assessments. Furthermore, despite the remote format, educators prioritized maintaining students’ training quality as well as their interest and commitment. Educators also had difficulty adapting to the available information and communication technology, as many professors experienced some trouble mastering this mode of teaching (7,8,11).

Regarding the practical activities that make up most of the courses in the final years of the three undergraduate programs, at first, the outpatient and hospital activities were either temporarily suspended or reorganized. Internet-based projects, online clinical case discussions, and online discussions between professors, supervisors, and students on pandemic-related issues were part of the emergency education restructuring. Their practical internship activities were suspended and postponed (11) until after the pandemic is under control. Part of the professors’, supervisors’, and students’ academic activities were reassigned to train other professionals and/or to care for the health of patients hospitalized with COVID-19 or other serious health conditions. Students were trained to address the biosafety and health care needs of COVID-19 patients.

It is important to highlight that both the University of São Paulo and the FMUSP developed various courses and tutorials to help the faculty handle educational tools and platforms, contributing to the quality of classes taught during the pandemic. In addition, the Graduation Dean at USP implemented strategies and actions to make course deadlines and requirements more flexible. This enabled undergraduate programs to continue, even if some courses, especially practical ones, were not concluded owing to social isolation. The graduation schedule was also changed to reflect these adaptations.

Concerning the remote pedagogical activities and the legal issues, an important aspect to point out is that, in terms of digital access, there is a huge difference between having electronic devices and an internet service, and being able to use both to follow the activities proposed by the university and professors. There is not yet a norm or guideline from MEC that instructs and establishes which format(s) are to be followed by the academic institutions. Academic institutions and their faculty are adapting and using increasingly more digital tools and platforms. For this essential teaching and learning process to be developed the best way possible, schools must provide communication and information channels between students and educators (12).

The changes that took place in recent months required a speedy adjustment on the part of the professors and students. They also raised questions about the lack of interactivity between students and professors as well as the overload of activities experienced by them. Other debates question how prepared professors of in-person courses are to teach in a format other than the traditional one and the factors that motivate them when carrying out RL.

Given all these challenges to the education system imposed by the COVID-19 pandemic, both students and professors feel that there are still many unanswered questions that need to be investigated. Among the main issues is the comparative value of learning acquired in RL *versus* in-person learning as well as the satisfaction of students and professors. Moreover, it is essential to mention the advances obtained over a short period of time, which can be incorporated into new hybrid routines. These achievements highlight the possibility of offering academic activities remotely, using online simulators, and deepening the professors' and students' knowledge of telehealth, communication tools, and information technologies.

## AUTHOR CONTRIBUTIONS

All authors participated in the discussions and writing of the manuscript and reviewed the manuscript in its final form.
